# A Bayesian Account of Visual–Vestibular Interactions in the Rod-and-Frame Task

**DOI:** 10.1523/ENEURO.0093-16.2016

**Published:** 2016-11-03

**Authors:** Bart B.G.T. Alberts, Anouk J. de Brouwer, Luc P.J. Selen, W. Pieter Medendorp

**Affiliations:** 1 Donders Institute for Brain, Cognition, and Behaviour, Radboud University Nijmegen, Nijmegen, The Netherlands; 2 Centre for Neuroscience Studies, Queen’s University Kingston, Kingston, Canada

**Keywords:** Bayesian inference, internal models, multisensory integration, rod-and-frame task, spatial orientation, verticality perception

## Abstract

Panoramic visual cues, as generated by the objects in the environment, provide the brain with important information about gravity direction. To derive an optimal, i.e., Bayesian, estimate of gravity direction, the brain must combine panoramic information with gravity information detected by the vestibular system. Here, we examined the individual sensory contributions to this estimate psychometrically. We asked human subjects to judge the orientation (clockwise or counterclockwise relative to gravity) of a briefly flashed luminous rod, presented within an oriented square frame (rod-in-frame). Vestibular contributions were manipulated by tilting the subject’s head, whereas visual contributions were manipulated by changing the viewing distance of the rod and frame. Results show a cyclical modulation of the frame-induced bias in perceived verticality across a 90° range of frame orientations. The magnitude of this bias decreased significantly with larger viewing distance, as if visual reliability was reduced. Biases increased significantly when the head was tilted, as if vestibular reliability was reduced. A Bayesian optimal integration model, with distinct vertical and horizontal panoramic weights, a gain factor to allow for visual reliability changes, and ocular counterroll in response to head tilt, provided a good fit to the data. We conclude that subjects flexibly weigh visual panoramic and vestibular information based on their orientation-dependent reliability, resulting in the observed verticality biases and the associated response variabilities.

## Significance Statement

Sensing the direction of gravity is very relevant for human perception and action. Although estimating gravity direction is known to depend on our inertial sensors, such as the vestibular organs, panoramic vision may also be important, providing cues that are oriented along gravity. The present study is the first that psychophysically characterizes this multisensory interaction. We further show that a Bayesian model involving a flexible weighting of vestibular and panoramic visual signals, with separate weights for vertical and horizontal visual cues, can account for the results. We discuss how this model could serve as a useful tool to establish the quality of signals in neurological disease.

## Introduction

Perception of upright requires integration of multiple information sources, including visual, vestibular, and somatosensory (Angelaki and Cullen, 2008; De Vrijer et al., 2008; Tarnutzer et al., 2009, 2010; Clemens et al., 2011). The subjective visual vertical task, in which roll-tilted subjects are asked to indicate the orientation of a line with respect to gravity, is often used to measure verticality perception. In the absence of panoramic information, subjects’ perception is accurate when upright, but biased when tilted. Roll tilt is overestimated for small roll angles (<60°, E-effect; Müller, 1916) and underestimated for larger roll tilts (>60°, A-effect; Aubert, 1861; Mittelstaedt, 1983; Mast and Jarchow, 1996; Jarchow and Mast, 1999; Van Beuzekom and Van Gisbergen, 2000; Van Beuzekom et al., 2001; Kaptein and Van Gisbergen, 2004; De Vrijer et al., 2008; Vingerhoets et al., 2008; Tarnutzer et al., 2009, 2010; Clemens et al., 2011).

Panoramic visual cues affect these biases (Mittelstaedt, 1986, 1988; Vingerhoets et al., 2009), for example, when the line is surrounded by a square frame (Asch and Witkin, 1948). When seated upright, biases in the rod-and-frame task show a cyclical modulation, with near-zero biases for upright and roll-tilted ±45° frame orientations. Biases are in the direction of the frame for in-between frame orientations (Wenderoth, 1973; Coren and Hoy, 1986; Spinelli and Antonucci, 1991; Zoccolotti and Antonucci, 1992; Zoccolotti et al., 1993; Spinelli et al., 1995; Bagust, 2005).

A square frame is not essential: a single peripheral line results in similar rod-and-frame effects (RFEs; Li and Matin, 2005a). This suggests that frames and single lines are fourfold gravity indicators: two related to the actual orientation and two perpendicular to it. This may be a remnant from a primitive global visual mechanism interpreting visual contextual cues as ambiguous head-in-space orientations, which can be combined with a vestibular head-in-space signal to determine the orientation of the head relative to gravity (Matin and Li, 1995; Li and Matin, 2005a, 2005b).

Previous studies supported this view by showing that the RFE decreases for larger frames, as if frame reliability as verticality indicator reduced (Ebenholtz, 1977; Ebenholtz and Glaser, 1982; Coren and Hoy, 1986; Antonucci and Fanzon, 1995; Zoccolotti et al., 1993; Spinelli et al., 1995). Similarly, roll-tilting the head increases the RFE, as if vestibular reliability of verticality reduced (Asch and Witkin, 1948; Witkin and Asch, 1948; Bischof and Scheerer, 1970; Benson et al., 1974; Goodenough and Oltman, 1981; DiLorenzo and Rock, 1982; Zoccolotti and Antonucci, 1992; Corbett and Enns, 2006; Dyde et al., 2006).

To account for visual–vestibular interactions, Vingerhoets et al. (2009) introduced a Bayesian inference model in which the RFE depends on statistical properties of the various signals involved. They computed a Bayesian estimate of gravity direction, optimally combining head-in-space cues from noisy visual-frame and vestibular information, and the *a priori* notion that head roll tilts are usually small. The frame contribution was conceived as a distribution of four equally probable head-in-space orientations. This model could account for the cyclic RFE modulation and increased biases at larger head-in-space orientations.

However, as Vingerhoets et al. (2009) pointed out, their model could not account for all data characteristics. First, with the head upright, the model predicts maximum frame influence at ±22.5°, because all four cardinal frame axes contribute equally to the upright percept. However, behavioral results show peak influence for frame orientations between 15° and 20°, suggesting nonequal contributions of the cardinal axes. Second, their model does not account for E-effects arising from uncompensated ocular counterroll (Palla et al., 2006; De Vrijer et al., 2008; Clemens et al., 2011) or changes in visual frame reliability. Moreover, because Vingerhoets et al. (2009) collected their data using an adjustment task, they had no appropriate measure of response variability, which led to model-fitting complexities.

Here, we refined their model to resolve these issues and tested it on novel psychometric data. The model was extended with factors to weigh the cardinal frame axes, account for visual reliability changes, and include uncompensated ocular counterroll. We collected rod-and-frame data in three conditions: baseline, reduced visual frame reliability, and reduced vestibular reliability.

Results show a cyclical RFE modulation, with zero biases for upright and roll-tilted ±45° frame orientations, and biases in the direction of the frame for in-between frame orientations. Furthermore, decreasing visual reliability reduced the RFE, whereas decreasing vestibular reliability increased the RFE. In all cases, we show that the refined Bayesian model describes the observations better than the original.

## Materials and methods

### Subjects

Nine subjects (seven female and two male, age 27 ± 5 years) without neurological disorders and with normal or corrected-to-normal vision participated in the study. All subjects received careful instructions about the experiment, after which they provided written informed consent. Subjects did not receive feedback about their performance.

### Setup

Subjects were seated in a height-adjustable chair such that their naso-occipital axis coincided with the midpoint of an OLED TV screen (LG 55EA8809, 123 × 69 cm, 1920 × 1080 pixels, refresh rate 60 Hz) in front of them. A height-adjustable chin rest supported the head in a natural upright position. An adjustable head cushion was used to support the head in a 30° orientation (right ear down) while the body remained upright. Head-in-space orientation was monitored several times per session using an angle-meter. Experiments took place in complete darkness.

### Experimental procedure

**Figure 1.**
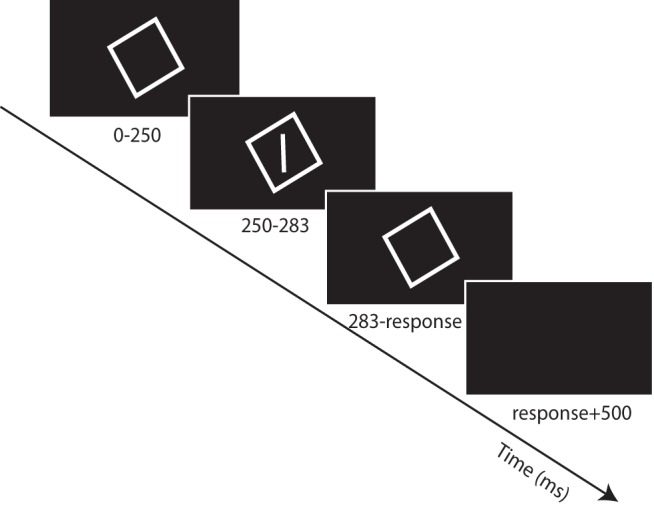
Experimental procedure of the rod-and-frame task. After presenting a square frame for 250 ms, a rod is briefly (33 ms) flashed within the frame. When the rod disappears, the square remains visible until the subjects responds whether the rod was rotated CW or CCW from upright. A 500-ms black screen is presented before the start of a new trial.

Figure 1 provides a schematic illustration of the task, which consisted of three different conditions (described below). Stimuli were presented in gray on a black background. Each trial started with presenting a square frame of 18.3 × 18.3° visual angle (31.5 × 31.5 cm), with a line width of 0.2° visual angle. The frame was displayed in an orientation randomly chosen from a set of 18 angles between –45° and 40° in intervals of 5°. After 250 ms, a luminous rod (angular subtense 12.6°) was briefly flashed (two frames, i.e., ∼33 ms) in the center of the frame. The rod orientation was randomly selected from a set of nine rod orientations centered around the gravitational vertical (–7°, –4°, –2°, –1°, 0, 1°, 2°, 4°, and 7°). Subjects indicated whether they perceived the orientation of the rod as rotated clockwise or counterclockwise from the gravitational vertical, by pressing the right or left arrow key respectively. Subjects were asked to respond as quickly as possible. After the response, the screen turned black for 500 ms and the next trial started. Trials were presented pseudorandomly, with each set containing one repetition of each combination of frame and rod orientation. In total, 10 sets were tested, yielding 1620 trials per condition.

This experimental procedure was used in three different conditions. (1) The baseline condition served to reproduce the RFE found in the original rod-in-frame experiments (e.g., Witkin and Asch, 1948), but now by using a psychometric procedure (Fig. 1). Subjects were seated 95 cm in front of the screen, and the frame and rod were presented with a luminance of 0.23 cd/m^2^. This condition served as a baseline for the other conditions: visual and vestibular. (2) The visual condition served to investigate the effect of a decrease in visual contextual reliability as an indicator of upright on the RFE. We reduced the retinal size of the rod and frame (by increasing the viewing distance of the display) to shift from peripheral stimulation (in the baseline condition, >10° visual angle) to parafoveal stimulation (i.e., <10° of visual angle). This alteration is known to reduce the RFE (Cian et al., 1995), as if there were less weight (i.e. more noise in Bayesian terms) of the frame on head-in-space orientation. We increased viewing distance from 95 to 224 cm, such that the square frame and rod had a visual angle of 8° × 8° and subtense of 5.4°, respectively. (3) In the vestibular condition, subjects rested their head against an adjustable head cushion mounted such that the head-on-body orientation was 30° right-ear-down (RED). Because it is known that head-on-body tilt changes the percept of the vertical (Aubert, 1861; Kaptein and Van Gisbergen, 2004; De Vrijer et al., 2008; Tarnutzer et al., 2010; Clemens et al., 2011), we first measured this percept without the presence of a frame. Subjects were presented with 10 sets of 11 randomly ordered rod orientations (–14°, –10°, –7°, –4°, –2°, 0, 2°, 4°, 7°, 10°, and 14°) centered around the gravitational vertical, yielding a total of 110 trials. Subjects had to indicate whether the orientation of the rod was rotated clockwise or counterclockwise from the gravitational vertical. We used this perceived orientation of gravity as the orientation relative to which the rod orientations (–10°, –7°, –4°, –2°, 0, 2°, 4°, 7°, and 10°) were presented in the rod-and-frame task. The RFE was tested with the head 30° RED, using the same experimental procedure as in the other conditions, but with the adjusted rod orientations.

### Data analysis

Clockwise frame and rod orientations were defined as positive. For each frame orientation, the proportion of clockwise responses as a function of rod orientation was examined. A psychometric curve was fitted through these data using a cumulative Gaussian function in Matlab (Wichmann and Hill, 2001):

(1) $$P\left(CW\right)=\lambda +\left(1-2\lambda \right)\frac{1}{\sigma \sqrt{2\pi }}\int_{-\infty }^{x}e^{-(y-\mu {)}^{2}/2\sigma^{2}}dy,$$

in which *x* represents the rod orientation in space and λ the lapse rate, accounting for individual stimulus-independent errors. The mean µ of the Gaussian and the standard deviation σ of the Gaussian account for subjects’ perceived orientation of gravity (i.e., the bias) and response variability, respectively. Fitting was performed adopting the method of maximum likelihood estimation using the Matlab routine “fminsearch.”

### Sensory integration model

To provide a theoretical framework that can explain the observed bias and variability of the RFE in the three conditions, we designed a Bayesian optimal integration model based on previous work of Vingerhoets et al. (2009). The Vingerhoets model consisted of an optimal integration of visual context, vestibular information, and prior knowledge about the head-in-space orientation. The current model was refined by accounting for manipulations in vestibular and visual reliability. The contribution of the visual contextual frame information was further refined by accounting for different sensory weights for the observed horizontal and vertical cardinal directions of the frame. We also extended the model with a step to account for ocular counterroll in response to head tilt.

#### Sensory input

**Figure 2.**
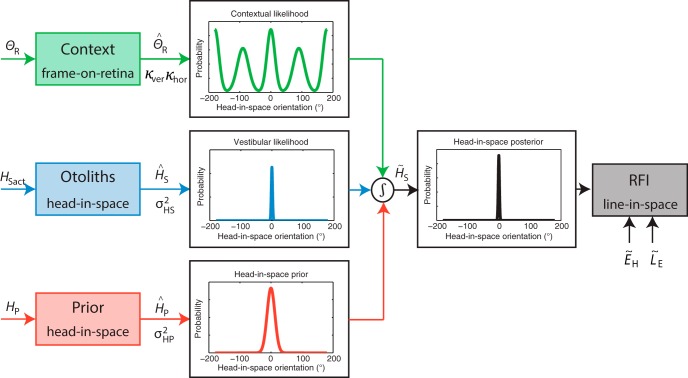
Schematic representation of the refined Bayesian optimal integration model for visual context. Physical signals about the retinal frame orientation ($\theta_{R}$), true head-in-space orientation (*H_Sact_*), and prior knowledge about likely head orientations (*H_P_*) are transformed into sensory signals, denoted by the hat symbol (ˆ). Sensory signals are assumed to be accurate but contaminated with Gaussian noise (κ, σ*_HS_*, and σ*_HP_*, respectively). For an optimal estimate of head-in-space orientation, denoted by a tilde (˜), the model integrates the contextual likelihood $P\left({\hat{\theta }}_{R}|H_{S}\right)$ together with the vestibular likelihood $P\left({\hat{H}}_{S}|H_{S}\right)$ and the head-in-space prior $P\left(H_{S}\right)$ This translates into multiplying the individual probability distributions: P(H˜S|H^S,θ^R)=P(H^S|HS)∗P(θ^R|HS)∗P(HS) The maximum of the resulting posterior distribution (MAP) is selected as the perceived head-in-space orientation (${\overset{˜}{H}}_{S}$), whereas the width of the curve is a measure of the response variability. The perceived orientation of the line in space is then obtained by a coordinate transformation using the eye-in-head orientation (${\overset{˜}{E}}_{H}$, uncompensated ocular counterroll) and the retinal rod orientation estimate (${\tilde{L}}_{E}$ , assumed to be veridical). The probability distributions in the figure represent the case in which the subject is seated upright (*H_S_* = 0°) with the frame displayed upright (θ*_R_* = 0°).

The model is schematically shown in Figure 2, in which the actual physical signals arriving at the sensory level are presented on the left, with capital letters indicating the physical signal and subscript indices the frame of reference. The sensors transform the physical signals into sensory signals, denoted by a hat symbol (ˆ). Optimal estimates are denoted by a tilde (˜). To obtain an estimate of the head-in-space orientation, the brain can directly use the information from the otoliths (${\hat{H}}_{S}$), which is presumed to be unbiased. This yields a vestibular likelihood function, $P\left({\hat{H}}_{S}|H_{S}\right)$, which is modeled by a Gaussian centered at the true head-in-space position, $H_{Sact}$, with standard deviation $\sigma_{HS}$ . Previous research has shown that the sensitivity of the otoliths decreases with larger head-in-space orientations (Rosenhall, 1972, 1974; Fernandez and Goldberg, 1976). Following Vingerhoets et al. (2009), this tilt-dependent decrease in sensitivity is accounted for by linearly increasing the noise of the otoliths as a function of head-in-space orientation:

(2) $$\sigma_{HS}=\alpha_{HS}|H_{Sact}|+\beta_{HS},$$

in which $\alpha_{HS}$ is the proportional increase of the noise level and $\beta_{HS}$ is the noise level when seated upright. In addition to direct head-in-space information, the model further assumes that the brain uses prior knowledge that our head is usually upright in space [$P\left(H_{S}\right)$]. This prior knowledge is in line with previous work (Eggert, 1998; MacNeilage et al., 2007; De Vrijer et al., 2008; Vingerhoets et al., 2009; Clemens et al., 2011) and modeled by a Gaussian centered on zero head tilt, with standard deviation $\sigma_{HP}$.

Furthermore, the brain can use panoramic visual cues from objects in the surrounding environment. For our specific experiment, we assume that the subject uses information from the four cardinal directions of the square frame. Following Vingerhoets et al. (2009), the model incorporates this as a sum of four von Mises distributions, with one peak at the observed frame orientation in retinal coordinates and the other peaks at 90° intervals:

(3) P(θ^R|HS)=∑i=14exp{κ(i)∗cos[θR+φ(i)−HS]}2π∗I0[κ(i)]

in which 2π is a normalization factor, φ denotes the four different cardinal directions of the frame (0°, 90°, 180°, and 270°), κ is the concentration parameter, and *I*_0_ is the modified Bessel function of the first kind with order zero. The concentration parameter κ of the four von Mises distributions is proportional to the inverse of the variance. In the model by Vingerhoets et al. (2009), it was assumed that this parameter was the same for all four peak locations. In the present model, we allow for dissociation of the variance of the cardinal axes of the frame. For convenience, we refer to the axis that is closest to the true gravitational vertical as vertical and the other axis as horizontal:

(4) $$\kappa \left(i\right)=\left[\kappa_{1},\kappa_{2},\kappa_{1},\kappa_{2}\right],$$

(5) $$\kappa_{1}=\kappa_{ver}-\left[1-\cos\left(\left|2*\theta_{R}\right|\right)\right]*\tau *\left(\kappa_{ver}-\kappa_{hor}\right),$$

(6) $$\kappa_{2}=\kappa_{hor}+\left[1-\cos\left(\left|2*\theta_{R}\right|\right)\right]*\left(1-\tau \right)*\left(\kappa_{ver}-\kappa_{hor}\right).$$

When the frame is not rotated and our head is upright in space (i.e., θ*_S_* = 0° and *H_S_* = 0°), the concentration parameter of the vertical orientations (φ = 0° and 180°) is set to κ*_ver_*, and the concentration parameter of the horizontal orientations (φ = 90° and 270°) to κ*_hor_*. When the frame is rotated, κ_1_ and κ_2_ change according to a cosine function such that their concentration parameters become equal at a frame orientation of θ*_S_* = ±45°. In this case, the contribution of all four cardinal axes of the frame to the head-in-space estimate is equal. The rate at which κ_1_ decreases and κ_2_ increases is determined by decline parameter τ, which has a value between 0 and 1. We used a gain factor *g* to control the relative variance between the baseline condition and the visual condition. It scales the variances ($\sigma^{2}\approx 1/\kappa$) of the visual contextual information in the baseline condition to the visual condition, such that gain factors >1 reflect an increase of the variance of the visual contextual signal and therefore a decrease in visual contextual reliability compared with the baseline condition.

Finally, the observed frame orientation in retinal coordinates ($\theta_{R}$) is given by

(7) $$\theta_{R}=-\left(\theta_{S}-H_{Sact}\right)-A_{OCR}*\sin\left(\left|H_{Sact}\right|\right),$$

in which $\theta_{S}$ is the actual frame orientation and *H_Sact_* is the true head-in-space orientation. Note that the observed retinal frame orientation has a sign opposite that of the true spatial frame orientation. After the central processing of head-in-space signals, the brain needs to transform the head-in-space signal into a line-in-space signal, because that is the coordinate frame in which the task is performed. This coordinate transformation is done by adding an eye-in-head estimate (the uncompensated ocular counterroll) and a line-on-eye estimate (assumed veridical). Previous visual context models (MacNeilage et al., 2007; Vingerhoets et al., 2009) did not incorporate uncompensated ocular counterroll, which has been suggested to play an essential role in explaining a bias away from head-in-space orientation in verticality perception (Palla et al., 2006; Clemens et al., 2011) Although ocular counterroll can be measured and is known to differ among subjects (Haustein and Mittelstaedt, 1990; Haustein, 1992), this bias reflects the part of ocular counterroll that is uncompensated, which is hidden, and can only be inferred (not measured). In our earlier work, we used Bayesian reverse engineering (Clemens et al., 2011) to infer this variable. Therefore, the present model incorporates eye torsion as $A_{OCR}*\sin\left(\left|H_{Sact}\right|\right)$, with parameter $A_{OCR}$ denoting uncompensated ocular counterroll.

#### Optimal integration

To obtain an optimal head-in-space estimate, Bayes rule indicates that all three probability distributions must be multiplied, which reveals the posterior distribution:

(8) P(H˜S|H^S,θ^R)=P(H^S|HS)∗P(θ^R|HS)∗P(HS).

The head-in-space orientation at which this posterior distribution has highest probability (i.e., the maximum *a posteriori*, or MAP) is what the brain assumes to be our head-in-space orientation. The MAP orientation is calculated using the expected value of the convolved signals. The width of the distribution is an indication of the variability of this measure, reflecting subjects’ response variability. The perceived orientation of the line in space is then obtained by a coordinate transformation using the eye-in-head orientation (uncompensated ocular counterroll) and the retinal orientation of the rod estimate (assumed to be veridical).

### Model fitting

The model consists of nine free parameters ($\alpha_{HS}$, $\beta_{HS}$, $\sigma_{HP}$, $A_{OCR}$, $\kappa_{ver}$, $\kappa_{hor}$, τ, *g*, and a lapse rate λ) that were fitted to the data. The model was first fitted simultaneously to the baseline and vestibular condition, to prevent the model from overfitting either the baseline or visual condition. The eight parameters that followed from that procedure were fixed in the second fitting to the visual condition only, in which the gain factor *g* was determined. The lapse rate λ accounts for individual lapses and was constrained to be smaller than 0.05 (5% of all trials). To prevent multiple solutions [combinations of prior knowledge ($\sigma_{HP}$) and ocular counterroll ($A_{OCR}$)] to explaining A-effects in the vestibular condition, we fixed $A_{OCR}$ to the previously reported value of 14.6° (Clemens et al. 2011).

Mean correction of the bias (McGuire and Sabes, 2009) was applied in the baseline and visual condition before model fitting to remove a systematic bias and asymmetries between clockwise and counterclockwise frame orientations, because the model assumes the data to be point symmetric around the frame orientation of 0°. Without mean correction, the model would overestimate variance to account for these asymmetries. In total, the eight free parameters had to account for 4860 stimuli and responses (3 conditions × 18 frame orientations × 9 line orientations × 10 repetitions). We fitted the model by maximizing the likelihood of the data in relation to these free parameters. Optimal parameters were obtained by minimizing the negative likelihood function using the Matlab routine “fmincon” (De Vrijer et al., 2008; Vingerhoets et al., 2009; Clemens et al., 2011). This routine was repeated three times with different initial starting values to make sure that the minimization procedure found a global minimum rather than a local minimum. Standard deviations of the fitted parameter values were obtained by performing 100 bootstrap runs. For each run, 4860 stimuli (reflecting the size of the dataset) and responses were randomly sampled with replacement from the raw data, keeping the amount of trials from each condition equal.

### Model evaluation

As a comparison to the refined model, we also fitted the model of Vingerhoets et al. (2009) including eye-torsion and a gain factor *g*, which is a more restricted model with equal variances at all four observed cardinal locations. To compare the maximum likelihood estimates of the Vingerhoets model to those of the present model, we used the Bayesian information criterion (BIC). The test statistic is defined as −2log(L)+k∗log(n). In this equation, *L* is the likelihood of the data given the model, *k* is the number of free parameters, and *n* is the number of observations (4860 stimuli and responses). A model with a lower BIC value refers to a more appropriate model. We furthermore compared the BIC values of the refined model to a purely descriptive model. The latter is based on a separate fit for each psychometric curve (with the bias and the slope of the curve as a free parameter), and a global lapse rate per subject. This results in a model with 109 free parameters (18 frame orientations × 3 conditions × 2 # free parameters + 1 lapse rate).

## Results

### Psychometric results

**Figure 3.**
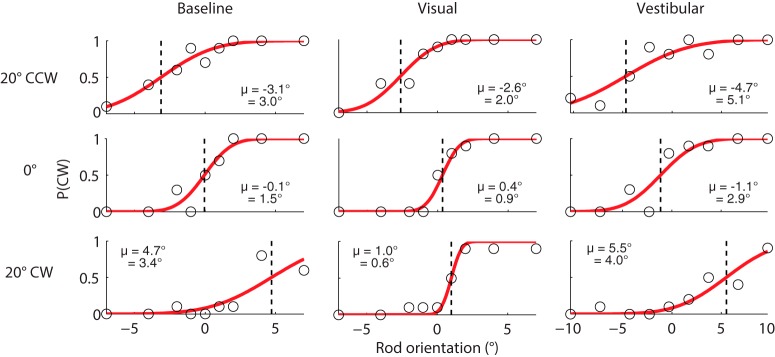
Probability of CW responses plotted against rod orientation for three example frame orientations (20° CCW, upright, and 20°CW) in a representative subject (black circles). Red solid lines represent the psychometric fits that quantify the bias (µ, dashed line) and variability (σ, inversely related to the slope) of the subject in each panel.

Figure 3 shows raw data of a representative subject in all conditions as the proportion of clockwise (CW) responses at each rod orientation for three exemplar frame orientations: 20° counterclockwise (CCW), 0°, and 20° CW in space. In all conditions and for all frame orientations, large CCW rod orientations (–7° for baseline and visual condition and –10° for vestibular condition) yielded low probabilities of responding clockwise, whereas large CW orientations (7° and 10°) yielded high probabilities of responding clockwise. Nevertheless, the distribution of responses is different for the different frame orientations, and across conditions. To quantify the bias and response variability of these distributions, we fitted psychometric curves to the three panels (red solid lines; see Methods).

With an upright orientation of the rod and frame, an ideal, unbiased subject would give 50% CW responses, reflecting a 0° bias. Indeed, in all conditions, biases are near zero when the rod and frame are not rotated (see dashed lines, µ = 0.05°, 0.35°, and –1.14°, respectively). Note that the small offset from zero in the vestibular condition is likely related to tilting the head on top of the body.

When the frame is rotated to ±20°, the perceived gravitational vertical shifts in the direction of the rotated frame, again in all conditions. Compared with the baseline condition, the visual condition produces smaller shifts of the bias when the frame is rotated, whereas the vestibular condition produces larger shifts of the bias. The slope of the psychometric curves quantifies the response variability, σ, at each frame orientation. In all three conditions, the slope is steeper for the upright frame than the ±20° rotated frame; hence, this subject shows a lower response variability at 0° frame orientation (σ is smaller). Note that the variability increases in the vestibular condition, indicative of the increase in vestibular noise with larger head-in-space orientations.

**Figure 4.**
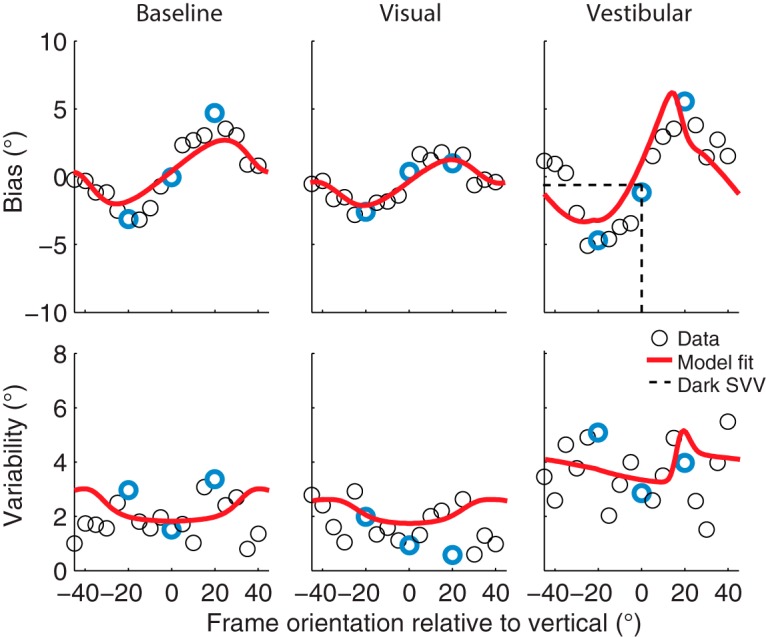
Bias and response variability plotted against frame orientation in a representative subject (circles) for all conditions. The biases and variabilities obtained from Figure 3 are highlighted in blue. Red solid lines represent the best fit of the Bayesian optimal integration model (Table 2). The dashed lines in the vestibular bias plot indicate the dark subjective visual vertical task (SVV).

Figure 4 shows the bias and variability plots of the representative subject for all frame orientations in each condition. The bias and variability measures of the example psychometric curves in Figure 3 are highlighted in blue. The bias pattern in the baseline condition confirms the shift in perceived gravitational vertical in the direction of frame orientation, peaking at a frame orientation of about 15–20°, and leveling off again for larger frame orientations. In addition, the subject shows a reduction in RFE in the visual condition and an increased RFE in the vestibular condition. The bias without the presence of the frame (dark subjective visual vertical task) is dashed in the vestibular condition and shows no substantial offset from zero. The response variability pattern shows an increasing variability, with larger frame orientations in the baseline and visual condition. In the vestibular condition, the overall response variability is higher relative to these conditions and becomes asymmetric owing to head tilt.

### Model predictions

The solid lines in Figure 4 are the best model fits of the Bayesian optimal integration model presented in Figure 2. The model captures both the bias and variability quite well in this subject. Best-fit parameters and their bootstrapped-based standard deviations are listed in Table 1 (S3).

**Table 1.** Best-fit parameters and bootstrap-based SD values

Subject	*σ_HP_*, °	β*_HS_*, °	α*_HS_*, °/°	σ*_ver_*, °	*σ_hor_*, °	τ	*g*	λ
S1	9.02 ± 0.51	2.22 ± 0.15	0.08 ± 0.01	6.23 ± 1.12	39.35 ± 18.43	0.83 ± 0.12	3.05 ± 0.75**	0.01 ± 0.00
S2	6.35 ± 3.65	2.30 ± 0.65	0.12 ± 0.05	6.91 ± 1.78	34.17 ± 22.57	0.96 ± 0.07	0.93 ± 0.60	0.08 ± 0.08
S3	8.16 ± 0.59	2.62 ± 0.31	0.09 ± 0.01	3.48 ± 0.76	104.58 ± 44.93	0.66 ± 0.02	1.31 ± 0.10**	0.03 ± 0.02
S4	4.44 ± 0.29	1.80 ± 0.15	0.07 ± 0.02	1.76 ± 0.92	37.86 ± 19.06	0.72 ± 0.10	1.38 ± 0.85**	0.01 ± 0.01
S5	9.39 ± 1.14	2.18 ± 0.20	0.11 ± 0.02	10.23 ± 2.72	55.52 ± 36.16	0.91 ± 0.12	0.67 ± 0.07**	0.02 ± 0.01
S6	4.52 ± 0.34	2.14 ± 0.15	0.03 ± 0.01	4.46 ± 0.73	41.11 ± 10.11	0.74 ± 0.07	1.25 ± 0.17**	0.02 ± 0.01
S7	4.28 ± 0.24	2.07 ± 0.31	0.04 ± 0.01	2.92 ± 1.52	69.47 ± 40.21	0.70 ± 0.12	1.22 ± 0.53**	0.01 ± 0.01
S8	6.09 ± 0.30	2.54 ± 0.22	0.08 ± 0.01	4.47 ± 0.75	58.08 ± 19.32	0.72 ± 0.06	1.02 ± 0.06**	0.02 ± 0.01
S9	6.29 ± 0.40	2.00 ± 0.18	0.02 ± 0.01	3.32 ± 1.28	30.19 ± 8.83	0.93 ± 0.10	0.99 ± 0.24	0.01 ± 0.00
All	6.50 ± 1.96	2.21 ± 0.25	0.07 ± 0.03	4.87 ± 2.57	52.26 ± 23.43	0.80 ± 0.11	1.31 ± 0.69	0.02 ± 0.02

Gains: **p < 0.001, *p < 0.01.

**Figure 5.**
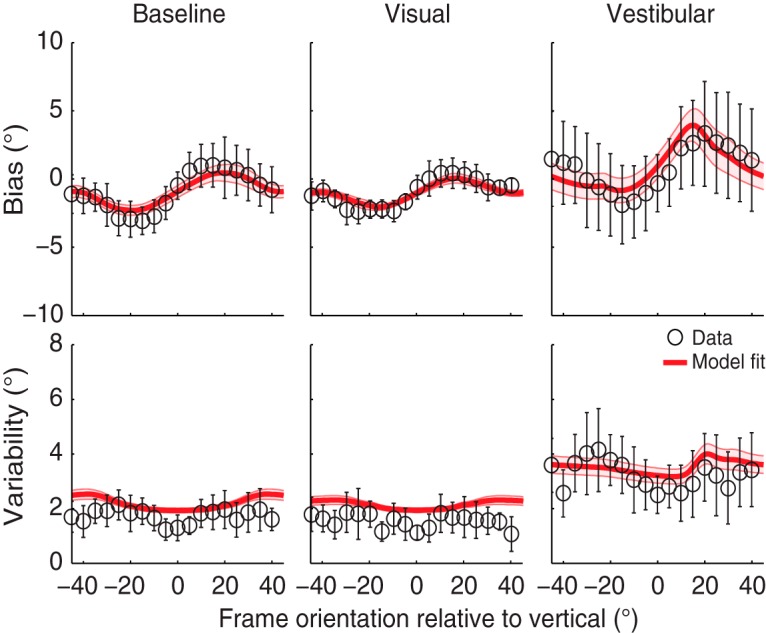
Mean bias and variability plots across all subjects for all conditions. Error bars represent the SD across subjects. The red solid lines on top of the data are the mean of the best fit across all subjects, with the shaded areas representing the SE on the model fit.

Figure 5 shows the mean bias and variability across the nine subjects, including the mean best fit of the optimal integration model fitted simultaneously to these data. Shaded areas indicate the standard error across the subjects’ best model fits. To test whether there is a significant effect of the visual condition, we performed a two-way paired univariate analysis of variance on the biases of the rod-and-frame task, with factors angle (5° to 40° in steps of 5°; we flipped and mirrored the CCW frame orientations) and condition (baseline vs. visual; Table 3). Results show a significant main effect of angle (F_(7,11)_ = 33.6, *p* < 0.001) and condition (*F*_(1,17)_ = 19.0, *p* < 0.001), and no interaction effect of angle on condition (*F*_(7,11)_ = 1.4, *p* = 0.31). Note that the bias is well captured by the fits of the optimal integration model (in red), in all three conditions. Despite some general overestimation, the model accounts reasonably well for the observed response variability, suggesting an increase of variability for larger frame orientations. Together, the results in Figure 5 show that the RFE and variability patterns are significantly influenced by the visual and vestibular manipulation and that these manipulations can be explained by a Bayesian optimal integration model of visual context, vestibular information and prior knowledge.

For each subject, best-fit parameter values and their bootstrap-based SD levels are listed in Table 1. Parameter $\alpha_{HS}$

is significantly larger than 0 (p < 0.001; Table 3), indicating that the vestibular noise increases when the head-on-body orientation is 30° RED. For reasons of clarity, we listed the SDs of the vertical and horizontal von Mises ($\sigma^{2}\approx 1/\kappa$) when the frame is not rotated. Variance in the vertical cardinal direction ($\sigma_{ver}$) is significantly smaller than in the horizontal cardinal direction ($\sigma_{hor}$; *p* < 0.001 for all subjects; Table 3), suggesting that subjects are more influenced by the vertical polarity of the frame than the horizontal. Gain factors are significantly larger than 1 for six of nine subjects, illustrating the reduction in RFE in the visual condition, whereas one of nine subjects shows a gain that is significantly lower than 1.

### Sensory weights

**Figure 6.**
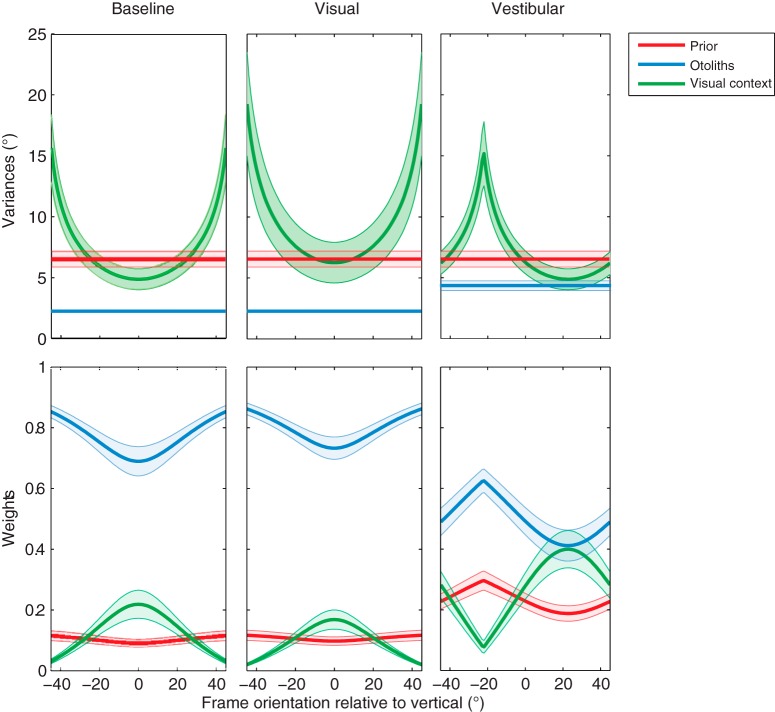
Prior (red), otoliths (blue), and visual contextual (green) weight distributions plotted against frame orientation in the different conditions. Shaded areas represent the SE across all subjects.

The top row of Figure 6 shows the mean variances of the prior knowledge (red), vertical visual context (green), and vestibular information (blue) across the different conditions. The mean is based on the fit results in Table 1. Shaded areas indicate the standard error across subjects. Note that the prior knowledge and vestibular variance are constant over frame orientation, with an increase in vestibular variance in the vestibular condition. By design, the optimal integration model assumes that the vertical visual context is lowest with an upright frame and increases with larger frame orientations in the baseline and visual condition. In the vestibular condition, the head is tilted 30° RED, which means that a perceived upright frame should be displaced 30° CCW. However, since the vertical visual context is processed in retinal coordinates, the lowest variance is found at a 30° CW frame orientation (see minus sign in Eq. 7). Note that this value is slightly off 30° CW because the uncompensated ocular counterroll (Eq. 7) shifts the distribution over frame orientation.

The sensory weights, indicating the relative contribution of the visual context, vestibular information, and prior knowledge, can be computed from these variances. The bottom row of Figure 6 shows their values for the three conditions. When the variance of the vertical visual context is low, the relative weight is high. This is reflected by a maximum contribution of visual contextual information in verticality perception of 15–25% when the frame is upright in the baseline condition. σ*_ver_* increases with larger frame orientations, which is illustrated by a decreasing sensory weight for the visual context and increasing weights for both the vestibular information and the prior knowledge at larger frame orientations. In the visual condition, the overall sensory weight for the vestibular information is slightly larger than in the baseline condition, whereas the overall weight for the visual information is slightly smaller than in the baseline condition. The opposite effect is seen in the vestibular manipulation condition, with a clear reduction in the vestibular weight. Note that in all conditions, the weight distribution is inversely related to the variances of the individual signals.

### Model evaluation

To test whether the assumption of different variances for observed vertical and horizontal cardinal directions is valid, we compared the present model to the original Vingerhoets model, assuming equal variances for both cardinal directions. We calculated maximum likelihood estimates of both models and corrected for the number of free parameters using BIC (Table 3).

We furthermore compared BIC values of our refined model to a purely descriptive account of the data by fitting separate psychometric curves to the data. Table 2 reports the BIC values of all models. The lowest BIC values, indicating a more appropriate model, are found for the refined Bayesian model in all subjects. To attribute the gain in effect size, we calculated Bayes factors from the difference in BIC values. All subjects have a Bayes factor larger than 20 when comparing the refined model to the Vingerhoets or psychometric model, which indicates that the refined model is decisive (Jeffreys, 1998).

**Table 2.** Model evaluation.

Subject	BIC refined model	BIC Vingerhoets model	BIC psychometric fits
S1	3114	3248	3764
S2	3524	3565	4141
S3	3649	3898	4092
S4	2736	2836	3089
S5	3487	3513	4184
S6	2486	2547	3180
S7	2926	3040	3526
S8	3568	3767	4161
S9	2743	2894	3340
All	3140 ± 434	3257 ± 460	3720 ± 447

## Discussion

In this study, we examined the interaction between vestibular and visual information in a rod-and-frame task in which subjects had to judge the orientation of a rod relative to the gravitational vertical. We quantified and compared subjects’ performance with psychometric measures of bias and variability at multiple frame orientations, in three conditions. In the baseline condition, we measured RFE with a 95-cm viewing distance and the head upright. In the visual condition, we decreased the visual reliability by increasing the viewing distance to 224 cm. In a vestibular condition, we decreased the vestibular reliability by tilting the head 30° on top of the body. In all three conditions, the RFE showed a cyclical modulation of perceived orientation of gravity, with near-zero biases for frame orientations close to the gravitational vertical or roll-tilted ±45°, and biases in the direction of the frame for in-between frame orientations. The magnitude of the RFE was reduced in the visual condition and enhanced in the vestibular condition. We furthermore found that variability was lowest when the frame was upright, increasing with larger frame orientations and leveling off again at ±45°. Overall variability was higher for the vestibular condition compared with both baseline and visual conditions.

We fitted a refined version of the optimal Bayesian integration model from Vingerhoets et al. (2009) to the individual subjects’ responses on each trial. This model was able to account for the bias and variability characteristics of the data in all three conditions (Fig. 5). It accounted for the difference in variance for the horizontal and vertical cardinal directions of the frame, and by doing so, it performed significantly better than the original model. The refined model also outperformed a purely descriptive model based on psychometric fits. Consistent with the model by Vingerhoets et al. (2009), the refined model also implies that vestibular information is weighted less when the head is tilted and more when visual information was made less reliable.

### Comparison with previous work

The present psychophysical findings on the cyclical modulation of the RFE in the baseline condition are in line with previous reports, in which subjects had to adjust a rod within a square frame to the gravitational vertical (Beh et al., 1971; Beh and Wenderoth, 1972; Wenderoth, 1973; Coren and Hoy, 1986; Spinelli and Antonucci, 1991; Zoccolotti and Antonucci, 1992; Zoccolotti et al., 1993; Spinelli et al., 1995; Bagust, 2005; Li and Matin, 2005b). We show that the strongest effects of the frame occur when the frame is tilted around 15–20°, indicating that participants are not simply influenced by the main axes of the frame, as previously suggested (Beh et al., 1971; Beh and Wenderoth, 1972). Rather, visual information is combined with vestibular information and prior knowledge that the head is usually close to upright.

The decrease and increase in magnitude of the RFE in the visual and vestibular conditions, respectively, confirm the results of previous studies in which the visual contextual reliability (Ebenholtz, 1977; Ebenholtz and Glaser, 1982; Coren and Hoy, 1986; Antonucci and Fanzon, 1995; Zoccolotti et al., 1993; Spinelli et al., 1995) or vestibular reliability (Asch and Witkin, 1948; Witkin and Asch, 1948; Bischof and Scheerer, 1970; Benson et al., 1974; Goodenough and Oltman, 1981; DiLorenzo and Rock, 1982; Zoccolotti and Antonucci, 1992; Corbett and Enns, 2006; Dyde et al., 2006) was manipulated. These observations are a clear indication of a visual–vestibular interaction that might be the origin of the RFE.

The novelty of our psychometric approach lies in quantifying this visual–vestibular interaction in the rod-and-frame task in both a vestibular and visual manipulation condition using an adequate assessment of the response variability. Variability of verticality perception has been addressed before using repeated measurements (“adjustments”; Haes, 1970; Mittelstaedt, 1983), or psychophysical assessment (Clemens et al., 2011; Alberts et al., 2016), and those studies showed that variability increases with larger roll tilts. As far as we know, variability has not been addressed before in rod-and-frame studies, which makes the present study the first to model both bias and variability using an inverse probabilistic analysis. This type of analysis has proven to be very successful in, for example, modeling bias and variability in verticality perception (De Vrijer et al., 2008; Clemens et al., 2011), as well as orientation perception within a surrounding visual context (Schwartz et al., 2006; Girshick et al., 2011; Wei and Stocker, 2015).

It is important to point out that the present model does not perfectly account for the data. For example, the fits show an overall overestimation of variability, particularly in the baseline and visual condition, and are not able to capture the peak variability at a frame orientation of ±30° (Fig. 5). This overall overestimation is the result of the fitting procedure, which reduces the fitting errors of the biases at the cost of response variability. By using symmetrized data, thus neglecting bias differences between clockwise and counterclockwise frame orientations that the model cannot explain, this effect disappears (not shown). Also, when fitting the model to one single condition instead of to all conditions simultaneously, it can capture the peak in variability better (not shown).

### Modeling aspects

We will now discuss how our current model relates to previous attempts in modeling the RFE. The basic architecture of the presented model, in which a noisy roll-tilt–dependent vestibular signal, prior knowledge, and a noisy panoramic visual cue are integrated, is very similar to previous modeling approaches of visual–vestibular interactions in spatial orientation (Mittelstaedt, 1986, 1988; Eggert, 1998; MacNeilage et al., 2007; Vingerhoets et al., 2009). However, to account for the characteristics in the data, we introduced three additional components to the model. First, to explain biases in the opposite direction of the head-in-space orientation in the vestibular condition, we incorporated uncompensated ocular counterrolling of the eyes (*A_OCR_*). Second, we argued that the two vertical cardinal axes of the frame provide us with more reliable cues about the gravitational vertical than the horizontal cardinal axes. We finally assumed that this relation between the different cardinal axes of the frame changes in a cosine fashion with frame orientation. Are these assumptions warranted?

It has been shown before that the eyes counterroll in the orbit when the head is tilted to a head-in-space orientation different from upright, peaking at ±90° roll-tilt (de Graaf et al., 1992; Markham and Diamond, 2002; Palla et al., 2006). Following previous work by Palla et al. (2006) and Clemens et al. (2011), we approximated ocular torsion with a sinusoidal function with a magnitude of 14.6°. It is known, however, that uncompensated ocular counterroll is subject dependent (Clemens et al., 2011). Because the head-on-body roll tilt is only 30° (i.e., not a whole range of head tilts), our Bayesian model cannot resolve the verticality bias in terms of prior knowledge of the head being upright or a combination of uncompensated ocular counterroll and prior knowledge. Ideally, we should have measured more and larger tilt angles, but this would not have been comfortable for the subjects. Given the data, we therefore fixed the magnitude of uncompensated ocular counterroll to a value previously reported (Clemens et al., 2011) because it adds only a linear shift over frame orientation at the output stage of the model (as also seen in Fig. 5), assuming this interferes only minimally with the optimal integration of visual context, vestibular, and prior information.

To validate these assumptions, we examined the refined model with the magnitude of uncompensated ocular counterroll free to vary between 0° and 15°, which is the range of ocular counterroll observed (Palla et al., 2006). This analysis revealed Bayes factors <20 in eight of nine subjects, indicating that uncompensated ocular counterroll as a free-fitting parameter is not decisive for the goodness of fit of the model and only causes overfitting (Table 3). Simulations further show that OCR amplitude has a nonlinear, but only marginal, effect on the fitted width of the upright prior. Taking these results together, it can be stated with confidence that whether uncompensated ocular counterroll (or torsion) is fixed, fitted, or not even included in the model is not critical or central to the reported findings.

**Table 3.** Statistical table.

Line	Location	Parameter	Data structure	Type of test	Confidence interval
a	Table 1	α*_HS_*	Bootstrapped α*_HS_* parameter values, normally distributed	Within-subject *t*-test	See Table 1, column 3
b	Table 1	*g*	Bootstrapped *g* parameter values, normally distributed	Within-subject *t*-test	See Table 1, column 7
c	Table 1	σ*_ver_* and σ*_hor_*	Bootstrapped σ*_ver_* and σ*_hor_* parameter values, normally distributed	Within-subject paired *t*-test	See Table 1, columns 5 and 6
d	Table 2	BIC refined vs. Vingerhoets model	Negative log(likelihood) values of both models	Bayesian information criterion	Not applicable
e	Table 2	BIC refined vs. descriptive psychometric model	Negative log(likelihood) values of both models	Bayesian information criterion	Not applicable
f	Results	Visual vs. baseline biases	Biases for different frame orientations and conditions	2-way ANOVA	Not applicable
g	Discussion	OCR validation	Negative log(likelihood) values of both models	Bayes factor	Not applicable

One reason to assume that the visual contextual information provides gravitational cues through four cardinal axes is the overrepresentation of horizontal and vertical cues in natural scenes (van der Schaaf and van Hateren, 1996; Coppola et al., 1998). More recently, Girshick et al. (2011) showed that this overrepresentation is reflected in subjects’ internal contextual prior distributions, with significant peaks at the cardinal directions. This confirms our description of visual contextual information processing in a Bayesian observer model (see Eqs. 3–7). In addition, Wei and Stocker (2015) showed that a Bayesian observer model constrained by efficient coding links the likelihood function and prior knowledge of our model, and both are jointly constrained by the natural statistics of a scene. They further showed that an asymmetric likelihood function is able to account for biases away from the prior. Although an asymmetric head-in-space likelihood function could cause a similar effect as uncompensated ocular torsion in the current model, we do not directly see a (neuro)physiological reason to assume this asymmetry (Rosenhall, 1972, 1974; Fernandez and Goldberg, 1976).

Because the rod-in-frame task specifically targets the gravitational vertical, it may well be that the vertical axes of an upright frame are more important than the horizontal axes for the verticality-derived estimate of visual information. When the frame is rotated in roll direction, however, the cardinal axes move with the frame. At a frame orientation of ±45°, all cardinal axes provide an equal amount of information, in addition to the vestibular information, about the gravitational vertical. This decline in reliability of the vertical frame axes and increase of reliability of the horizontal axes is captured in the present model by a cosine tuning function of the noise associated with the different cardinal axes. Note that we added a decline parameter, τ, which determines the (subject-specific) rate at which the noise in the vertical cardinal axes decreases with frame orientation. The closer this value is to 1, the steeper the noise will increase.

In addition to the decline parameter, the model contains a gain factor, *g*. The gain factor scales the variances of the cardinal axes of the frame in the baseline condition to those needed to account for a decrease in visual reliability in the visual condition. This allows fitting of both the vestibular and visual manipulation at once. Table 1 shows that six of nine subjects have a gain that is significantly larger than 1, confirming the hypothesis that visual reliability is reduced in the visual condition. Recently, Tomassini et al. (2010) showed that when the variance of the contextual information increases, uncertainty about the orientation of visual context grows. This confirms the results of our visual condition in which the uncertainty of visual context as an indicator of upright changes when shifting from peripheral to parafoveal stimulation.

**Figure 7.**
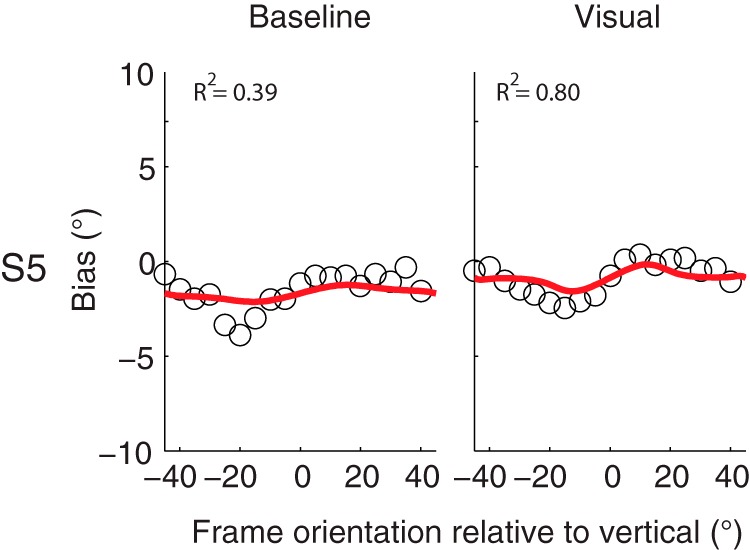
Bias plotted against frame orientation in the baseline and visual condition for subject S5. Red solid lines indicate the best fit of the Bayesian optimal integration model. *R* ^2^ values indicate the goodness of fit of the model to the data.

One subject, however, has a gain that is significantly lower than 1. When looking at the individual bias curves, this subject shows a higher peak-to-peak effect in the baseline condition (3.53°) relative to the visual condition (2.80°), which would correspond with lower visual reliability and a gain factor larger than 1. However, the RFE peaks at different locations for the baseline and visual condition, whereas in the model this location can only be the same, as it assumes linear scaling. The model fits show that it captures the visual condition perfectly, but underestimates the RFE in the baseline condition (Fig. 7). This is illustrated by the goodness-of-fit values (*R* ^2^ values) to the data in the baseline condition (*R* ^2^ = 0.39) relative to the visual condition (*R* ^2^ = 0.80).

### Neurophysiological implications

Previous accounts of the rod-and-frame task suggest that the visual–vestibular interactions found in the RFE arise from a rather primitive global visual system that interprets a visual contextual cue as an ambiguous head-in-space orientation signal, which is combined with nonvisual head-in-space orientation signals from the otoliths (Matin and Li, 1995; Li and Matin, 2005a, 2005b). Although it is known that the orientation of a simple two-dimensional object is processed in the early visual areas (V1+; Serre, 2014), only a few recent studies have looked at how this visual contextual information can be used in the subject’s perception of verticality. Two regions have been reported to play a role in the rod-and-frame effect.

Walter and Dassonville (2008) suggested a role for the right superior parietal lobule (rSPL). They used functional MRI to show that subjects had a higher activation in this area when locations needed to be judged relative to a visual context. In further support, Lester and Dassonville (2014) showed that stimulation of the rSPL increased the bias when judging the orientation of a rod within a tilted square frame. They further showed that stimulation of the rSPL did not increase biases in the tilt illusion, which means that the rSPL is not purely related to orientation processing, but is rather involved in higher cognitive processes such as the visual–vestibular interactions in the rod-and-frame task.

Other recent brain stimulation studies have established the causal role of the right temporoparietal junction (rTPJ) in estimating the visual vertical (Pérennou et al., 2008; Kheradmand et al., 2013; Fiori and Candidi, 2015). Fiori and Candidi (2015) showed that constant theta burst stimulation of the rTPJ significantly impaired the ability to establish the visual vertical, without a modulating effect of surrounding visual frame. This indicates that rTPJ is involved only in establishing an internal verticality percept, without weighting it with visual contextual information. Thus, whereas the SPL modulates the percept of verticality based on visual context, the TPJ seems to process signals related to an internal estimate of verticality. This interaction may suggest that the rTPJ and the early visual areas (V1+) have reciprocal inhibitory connections, which both project toward the rSPL where the RFE is processed. These inhibitory connections may reflect the sensory weighting described in Figure 6. Thus, when the visual cue becomes relatively more reliable (vestibular condition), the early visual areas might inhibit the rTPJ such that the percept of the vertical in the rSPL will be mainly based on the visual contextual cues.

### Clinical implications

**Figure 8.**
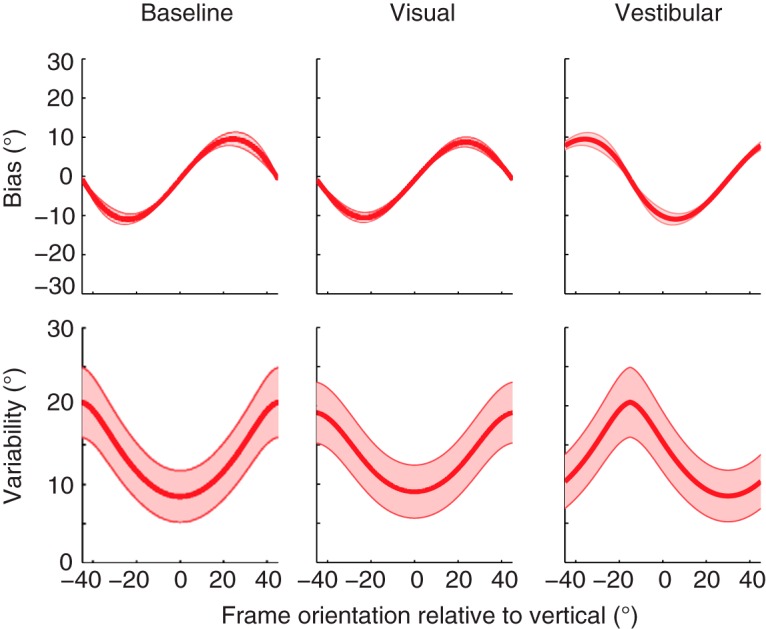
Simulations of the Bayesian optimal integration model for bias and response variability in patients with complete vestibular function loss. Shaded areas represent the SE on the model simulations.

The dynamic sensory weighting process underlying the RFE might be of particular importance for different patient groups. For example, previous research has shown that bilateral vestibular patients rely on sensory substitution for verticality perception. Figure 8 shows predictions from our refined model for the three different experimental conditions for bilateral vestibular patients. These simulations are based on the mean parameter values of Table 1, with the values for the vestibular reliability (α*_HS_*, β*_HS_*) set to infinity. The plot shows an increase in magnitude of the RFE up to about 10°, which corresponds to the results of previous research with bilateral vestibular patients (Guerraz et al., 2001). Interestingly, the model predicts that there will be no changes in the magnitude of the bias in the visual and vestibular manipulation condition.

Interesting patients to study with the present paradigm are those with higher-order vestibular disorders such as the room tilt illusion. These patients often experience upside-down vision or 90° tilts of the space, which is a clear indication of an error in verticality perception. This may arise at the level of the vestibular inputs, but could also arise from a lesion in parietal-occipital areas such as the rSPL (Sierra-Hidalgo et al., 2012). Indeed, a recent study argued that the room tilt illusion arises owing to a cortical mismatch of the visual and vestibular three-dimensional egocentric representation of verticality (Brandt et al., 2014), which is likely to be located in higher-level areas such as the rSPL.

## Conclusion

We have tested the performance of healthy subjects in a regular rod-and-frame task and two manipulations of this task. We showed that a Bayesian optimal integration model can fit the data and that the assumption of different variances for horizontal and vertical cardinal axes of the frame is warranted. We furthermore showed that the bias and variability of these subjects can be linked to a flexible weighting of visual and vestibular sensory signals. Finally, we coupled the presented model to neurophysiology and clinical populations, which makes the psychometric assessment of the RFE a useful tool to establish the quality of signals in neurological diseases.

## Synthesis

The decision was a result of the Reviewing Editor Kathryn Jeffery and the peer reviewers coming together and discussing their recommendations until a consensus was reached. A fact-based synthesis statement explaining their decision and outlining what is needed to prepare a revision is listed below. The following reviewers agreed to reveal their identity: Stefan Glasauer, Jean Laurens

The reviewers and myself have examined the revised version and we are pleased to say that we have found it suitable for publication in its present form, and would like to thank you for working with the reviewers to enhance the paper.

We would like to encourage you to submit a visual abstract, which explains your findings at a glance for a non-specialist reader.
